# An Efficient Organoid Cutting Method for Long-Term Culture and High-Throughput Analyses

**DOI:** 10.1007/s13770-025-00731-y

**Published:** 2025-06-16

**Authors:** Nicholas A. Chartrain, Marina V. Pryzhkova, Juliana I. Candelaria, Kristin H. Gilchrist, Philip W. Jordan

**Affiliations:** 1 4D Bio3 Center for Biotechnology and Department of Radiology and Bioengineering, Uniformed Services University of the Health Sciences, Bethesda, MD 20814 USA; 2 The Geneva Foundation, Tacoma, WA 98402 USA; 3 Department of Biochemistry and Molecular Biology, Uniformed Services University of the Health Sciences, 4301 Jones Bridge Rd, Bethesda, MD USA; 4 The Henry M. Jackson Foundation for the Advancement of Military Medicine, Bethesda, MD USA; 5 Department of Biochemistry and Molecular Biology, Johns Hopkins University Bloomberg School of Public Health, Baltimore, MD USA; 6 School of Biomedicine, The University of Adelaide, Adelaide, SA 5000 Australia

**Keywords:** Organoid, Human pluripotent stem cells, Bioengineering, Long-term culture, High-throughput

## Abstract

**Background:**

Human organoid models are invaluable for developmental studies, disease modeling, and personalized medicine research. However, long-term maintenance is challenging due to hypoxia and nutrient limitations as organoids grow. Cutting organoids improves viability, but current methods have low throughput and are prone to causing culture contamination. This study introduces an efficient organoid cutting method to enhance long-term culture and enable high-throughput analyses.

**Methods:**

We employed three-dimensional (3D) printing to fabricate four classes of organoid cutting jigs with blade guides that were compared and optimized for consistent sectioning of human pluripotent stem cell (hPSC)-derived organoids. Organoids were cultured in mini-spin bioreactors and cut every three weeks, beginning on day 35. Organoid health and growth were evaluated by size increase and proliferative marker expression. Additionally, we utilized 3D printed molds to create GelMA or Geltrex-embedded organoid arrays and silicone molds for optimal cutting temperature compound (OCT)-embedding of organoid arrays.

**Results:**

All 3D printed jigs enabled rapid and uniform organoid cutting under sterile conditions. We determined that a flat-bottom cutting jig design had superior cutting efficiency. Cutting improved nutrient diffusion, increased cell proliferation, and enhanced organoid growth during long-term culture. The mold-based approaches enabled the creation of densely packed organoid arrays and cryosections with evenly distributed organoids.

**Conclusion:**

This novel organoid cutting and arraying method overcomes limitations in long-term organoid culture and high-throughput processing. The simplicity of the cutter design and handling make it a versatile tool for diverse types of organoids. By enhancing organoid viability and enabling consistent sample preparation, this approach facilitates improved organ development and disease modeling, drug screening, and high-throughput analyses, including single-cell spatial transcriptomics applications.

**Supplementary Information:**

The online version contains supplementary material available at 10.1007/s13770-025-00731-y.

## Introduction

The development of organoid technology has revolutionized biomedical research by providing new, powerful *in vitro* models. These three-dimensional (3D) self-organized cell aggregates demonstrate the complex architecture of native tissues or organs and closely mimic their functions [1]. This technology has opened new avenues for studying human biology and disease in ways that are not possible using animal models due to species-specific differences. Organoids can be generated from patient tissues and human pluripotent stem cells (hPSCs) [1, 2], permitting new possibilities for personalized medicine and identifying effective treatment strategies. For example, tumor organoids have shown promise in predicting patient-specific drug responses [1]. Intestinal organoids from cystic fibrosis patients have been used to study disease mechanisms and test potential therapies [3, 4]. Similarly, liver organoids have been employed to model genetic liver disorders and investigate drug-induced liver injury [3].

hPSCs have unlimited proliferative and differentiation capabilities and can be generated for any individual using somatic cell reprogramming technologies [5]. Organoids generated from hPSCs can recapitulate human development in utero and provide a powerful platform for disease modeling and developmental studies [6, 7]. For example, brain organoids have been used to investigate the complex cellular interactions and molecular mechanisms underlying human brain development [8]. These organoid culture systems recapitulate key aspects of brain organization and cellular diversity, allowing for detailed analysis of neurogenesis, cortical layer formation, and regional specification [1, 6]. A highlighted benefit of this technology is the scalability of organoid culture, thus enabling screening of the effects of chemical exposures on human tissues. For example, human organoids that mimic airway, brain, liver, and cardiac tissue have been used to study the impact of environmental toxins on organ development and function [3]. This approach offers a more accurate representation of human tissue responses compared to traditional animal models or monolayer cell cultures.

Despite the potential that human organoid models hold for biomedical research, there are several challenges that limit their long-term viability and functionality [9]. hPSC-derived organoids utilized for developmental studies require extended culture to transition from embryonic to fetal and more advanced developmental stages [1]. During this time, organoids significantly increase in size, which creates hypoxic conditions and nutrient deprivation in the organoid core due to diffusion limits. This can lead to cell death and altered cellular behavior, compromising the ability of an organoid to model tissue function accurately. Cerebral organoids have been shown to develop a necrotic core as they increase in size, affecting their ability to model later stages of brain development [10]. These size-related issues are also the case for human tissue-derived organoids [11]. To address these challenges, researchers have developed various strategies to enhance organoid viability and functionality during long-term culture.

Mechanical and enzymatic dissociation are relatively simple techniques to decrease organoid size and increase the total number of organoids [12–14]. However, these methods compromise crucial cellular organization in complex organoids and can only be used for histologically simple tissues, such as epithelial organoids. Another approach to mitigate hypoxia and nutrient deprivation during long-term culture is to slice organoids into smaller pieces using a surgical scalpel under a dissection microscope [15, 16]. Alternatively, organoids embedded in agarose gel can be cut using a vibratome [10, 17]. These techniques have been successfully applied to cerebral organoids, allowing for extended culture periods and improved cellular diversity [10, 15]. Fragmenting organoids into smaller sizes can help maintain cell viability and functionality in the core regions by reducing the diffusion distance for oxygen and nutrients. While these methods have shown some success, they suffer from low throughput, require specialized equipment, or may compromise sterile culture conditions. Thus, novel techniques that mitigate necrotic core formation and enable long-term organoid culture are needed.

Downstream analysis of organoid structure and function is essential and, for many researchers, involves using sectioning techniques. Cryosectioning and microtomy are commonly used to prepare thin sections of organoids for morphological assessment, immunofluorescence microscopy, and RNA in situ hybridization. These techniques allow for a detailed analysis of cellular organization and gene expression patterns [18–20]. Vibratome sectioning enables the preparation of thicker organoid slices, which can be helpful for electrophysiological studies or live imaging experiments [10]. Unfortunately, current methods for sectioning tissue struggle with low efficiency and inconsistent placement of organoids, hindering bulk evaluation [21]. This can make it challenging to perform high-throughput analyses or consistently compare multiple organoid samples [22].

Developing methods to prepare and position organoids in a compact, uniform array would greatly enhance the efficiency of high-throughput assessments using standard techniques like immunofluorescence microscopy and RNA in situ hybridization. Furthermore, the ability to compact organoids together for analysis is particularly important for emerging technologies such as spatial transcriptomics, where every region of the slide is valuable in terms of cost and data capture [21, 23, 24]. Improved sectioning techniques would enable comprehensive analysis of organoid structure and function, facilitating deeper insights into human development and disease pathophysiology. Additionally, such advancements would support the scale-up of organoid-based drug screening and toxicology studies, accelerating the translation of organoid research into clinical applications.

In this study, we present an approach for cutting live organoids, which improves their proliferative capacity while also leading to more uniform size and shape. The simplicity of the cutter design and handling make it a versatile tool for diverse types of organoids. Utilizing hPSC-derived gonad organoids as an example, we showed that with regular cutting, organoids can be maintained in culture for approximately five months and undoubtedly beyond. We also developed a technique utilizing molds to make densely packed organoid arrays for cryosectioning. This facilitated consistent sample preparation suitable for high-throughput studies, including spatial transcriptomics. This work will benefit the field of organoid research and extend the limits of organoid culture and analysis.

## Materials and methods

### Design of organoid cutting jigs

Organoid cutting jigs, blade guides, and blade guard were designed using Autodesk Inventor Professional 2024. Digital models are available in .stl and .ipt formats for both direct use and modification (Supplementary files S1-S16). The .ipt and .stl design files have also been published as entry 3DPX-021856 on the NIH 3D database, an open database of bioscientific and medical models [25]. The cutting jigs, blade guides, and blade guard were 3D printed using BioMed Clear resin (Formlabs, Somerville, MA, USA) and a Form3B 3D printer (Formlabs). Parts were cleaned and post-processed according to the manufacturer's instructions and sterilized prior to use. Double-edge safety razor blades (Superior Platinum, Astra, Gillette India) were used for organoid cutting.

### Organoid culture

hESC (H1, WiCell, Madison, WI, USA) maintenance and differentiation were conducted as previously described [26]. hESC differentiation was performed through the formation of three-dimensional cell aggregates, known as embryoid bodies, which on day 6 (D6) of culture were transferred into a mini-spin bioreactor and allowed to develop into early male gonad organoids as described [26]. Mini-spin bioreactor setup and operation were performed as previously published [27, 28]. Organoids were first cut on D34-35 of culture and then every 3 weeks (± 3 days). Cut organoids were allowed to recover for 6 days before collecting for analysis, together with uncut (never cut) controls.

### Organoid cutting

The cutting of organoids was performed in a biosafety cabinet with pre-sterilized tools. Organoids were collected from the mini-spin bioreactor into a 50 mL conical tube containing DMEM/F12 with HEPES (Corning, Corning, NY, USA). About 30 organoids were aspirated in a small amount of medium using a cut 1000 µL pipette tip and deposited into the channel of the cutting jig base, which itself was placed in a 100 mm diameter cell culture dish, or on a sheet of polydimethylsiloxane (PDMS) in the case of the racetrack cutting jig. A 200 µL pipet tip was used to carefully remove excess medium from the channel. Sterile, fine-point tweezers were used to gently align the organoids so that each was at the bottom of the cutting jig channel without contacting adjacent organoids. The blade guide was then positioned onto the jig base. To slice the organoids, the blade was pushed down through the blade guide until it contacted the bottom of the cutting jig channel (or the PDMS sheet, in the case of the racetrack cutting jig). After the removal of the blade and blade guide, the cut organoids were flushed out with medium into a clean dish. The underside of the blade guide was checked for any stuck organoid halves, which were collected using sterile tweezers. Sliced organoids were collected into a new 50 mL conical tube. The cutting process was repeated for all organoids collected from the mini-spin bioreactor. Cut organoids were then returned to the bioreactor for continued culture.

### Generating organoid arrays for cryosectioning

#### Organoid arrays in GelMA

Organoids were fixed in 2.5% formalin in PBS for 20–30 min at room temperature (for organoids < 3 mm diameter) or for one hour (h) at 4 °C (for organoids > 3 mm diameter), washed in PBS, and incubated in 20% sucrose in PBS at 4 °C until saturation (~ 2–3 h). Organoids were placed into a 25 mL divided reagent reservoir (Thomas Scientific, Swedesboro, NJ, USA) using a cut 1000 µL pipette tip, and sucrose solution was removed using a 200 µL pipet tip. Organoids were carefully scooped up with a metal spatula, the remaining sucrose solution was removed using the edge of a Kimwipe (Kimberly-Clark, Irving, TX, USA), and organoids were placed into a 3D printed mold (Supplementary files S17-18) on a glass slide. GelMA (CellINK, Gothenburg, Sweden) mixture was warmed to 37 °C and pipetted into the mold to just cover the single layer of organoids. The GelMA/organoid mixture was UV irradiated from above for 60 seconds (s) using a handheld 405 nm LED UV lamp (F1RST Layer, Amazon, Seattle, WA, USA) and from below through the glass slide for 30 s. If larger blocks of organoids were desired, additional layers of organoids and GelMA were added, with UV irradiation applied after each layer, until the desired height was achieved. The mold was then lifted off the glass slide, and the polymerized GelMA/organoid block was gently pushed out of the mold. The GelMA/organoid block was placed in Fisher Healthcare Tissue-Plus OCT compound (Thermo Fisher Scientific, Waltham, MA, USA) in a peel-a-way embedding mold (Polysciences, Warrington, PA, USA), frozen at -80 °C, and cryosectioned.

#### Organoid arrays in geltrex

The square 3D printed mold was placed on a glass slide covered with parafilm and gently pressed to create a seal. The glass slide and mold were placed on ice. As detailed in Sect. 2.4.1, organoids were fixed in 2.5% formalin in PBS and saturated with 20% sucrose in PBS, then deposited into the mold, and the sucrose solution was removed. The organoids were mixed with non-diluted Geltrex (Gibco, Waltham, MA, USA) that was thawed on ice. The glass slide was then placed in a 37 °C incubator for 30 min to allow the Geltrex to polymerize. During incubation, a small amount of OCT compound was poured into an embedding mold to cover the bottom and placed at -80 °C or on dry ice. Once the Geltrex polymerized, the Geltrex/organoid block was gently pushed out of the mold. Then, with the embedding mold still on dry ice, a small amount of fresh OCT was added on top of the hardened OCT, and the Geltrex/organoid block was quickly placed on top of the fresh OCT. Additional OCT compound was poured around the Geltrex/organoid block to cover it. The embedding mold was then placed at -80 °C to freeze all the OCT. Samples were stored at -80 °C until cryosectioning.

#### Organoid arrays in silicone molds

Soft semi-clear silicone (1.5 mm thickness, Exactly Rubber, American Rubber Products, Santa Ana, CA, USA) was cut with a scalpel into the desired shape and size to form a mold and gently pressed down onto a glass slide covered with parafilm to create a seal. Organoids, fixed and cryoprotected as described in Sect. 2.4.1, were placed into the mold, and the sucrose solution was removed. Organoids were mixed with OCT compound within the silicone mold, and the glass slide was placed on dry ice to freeze the OCT compound. A small quantity of OCT compound was applied to the bottom of the embedding mold and frozen by placing the embedding mold on dry ice. The parafilm was gently peeled away from the frozen silicone mold with organoids and the mold was placed onto a small amount of fresh OCT deposited on the pre-frozen OCT layer (on dry ice). Additional OCT compound was poured around the silicone mold to cover it. The embedding mold was placed at -80 °C to freeze completely. Samples were stored at -80 °C until cryosectioning.

### Immunohistochemistry

OCT-embedded samples were cryosectioned at 16 μm thickness using the Epredia CryoStar NX70 (Thermo Fisher Scientific). Sections were mounted on Selectfrost Adhesion (Thermo Fisher Scientific) microscope slides and stored at -80 °C until further processing. Sections were first permeabilized and blocked with 0.25% Triton X-100 and 10% goat serum in PBS for 30 min at room temperature. Next, sections were incubated with the primary antibodies: Ki67 (#9129, 1:400, Cell Signaling Technology, Danvers, MA, USA), SOX9 (#82630, 1:300, Cell Signaling Technology), and collagen IV (#SAB4200709, 1:400, Sigma-Aldrich, Darmstadt, Germany) diluted in blocking solution (PBS with 10% goat serum) overnight at 4 °C. Then, slides were washed 3 times with rinse buffer (PBS with 0.05% Tween-20, Sigma-Aldrich) and incubated in blocking solution with secondary goat anti-rabbit or anti-mouse Alexa Fluor 568 or 488 antibodies (Invitrogen, Waltham, MA, USA) for 1.5 h at room temperature. After washing 3 times with rinse buffer and a final wash with PBS, slides were mounted with Vectashield Antifade Mounting Medium with DAPI (4',6-diamidino-2-phenylindole, Vector Laboratories, Newark, CA, USA).

### Live organoid staining

Dead cells in live organoids were stained with Live-or-Dye NucFix™ Red Staining Kit (Biotium, Fremont, CA, USA) according to the manufacturer’s instructions. Briefly, organoids were washed once with DMEM/F12 with HEPES (Corning) and incubated in the same medium with added Live-or-Dye stain (1:2000) for ~ 1 h at 37 °C in the CO_2_ incubator (5% CO_2_). After incubation, organoids were washed once with PBS and imaged in fresh DMEM/F12 with HEPES medium.

### Microscopy and imaging

Live cell and immunofluorescence image capture and analysis were performed using Keyence BZ-800 and BZ-X800 Viewer and Analyzer software (Keyence, Osaka, Japan). Adobe Photoshop (San Jose, CA, USA) and ImageJ (National Institutes of Health, Bethesda, MD, USA) were used to prepare figure panels. Organoid cutting jigs and blade guides were imaged with a Google Pixel 6 Pro (Google, Mountain View, CA, USA). Live organoids were imaged either in 12-well plates (Falcon #351143, Corning) or in 24-well plates (NEST #702003, NEST Scientific Wuxi, Jiangsu, China).

### Statistical and data analyses

Images of uncut control and sliced organoids were analyzed to determine the cutting efficiency of each jig. ImageJ was used to threshold and process images [29]. The 3D Objects Counter plugin [30] was used to quantify the number of pixels and calculate the size of each organoid. (μm^2^, Supplementary file S19).

The Keyence Macro Cell Count analysis software was used to quantify Ki67+ nuclei ratio in organoids. Ki67+ nuclei and total nuclei stained with DAPI were counted in a 200 × 200 μm field. At least 100 cells were counted per organoid, and 10 organoids were analyzed per sample. Two biological replicates were performed. For Ki67 localization analysis, the radius of D104 uncut and cut organoids was measured and divided into three compartments to define the length of the inner, middle, and outer regions of each organoid. Two perpendicular areas (200 μm width) were measured, and Ki67+ nuclei were counted and divided by the total number of cells in each region. Ten organoids were measured per sample with two biological replicates. The increase in organoid size during three consecutive cuttings from a single experiment was calculated by fold increase in the average organoid area using ImageJ.

Statistical analysis was performed using GraphPad Prism 10 software (San Diego, CA, USA). The comparison of two samples was performed using the Mann–Whitney test (Ki67+ field analysis), unpaired t-test (Ki67+ localization analysis), or one-way ANOVA with Dunnett’s multiple comparisons post hoc test (organoid cutting jig evaluation). Differences between samples were considered statistically significant when *p* values were < *0.05* (*), < *0.01* (**), and < *0.001* (***).

## Results

### Limitations of long-term organoid culture

hPSC-derived organoids resemble embryonic and fetal-like tissues and require long-term culture to advance to later developmental stages [1]. For example, human brain organoids need to be grown *in vitro* for 100 days to resemble the fetal brain at week 17–24 post-conception and 150 days to show similarity to a postnatal brain [10, 31].

**Fig. 1:**
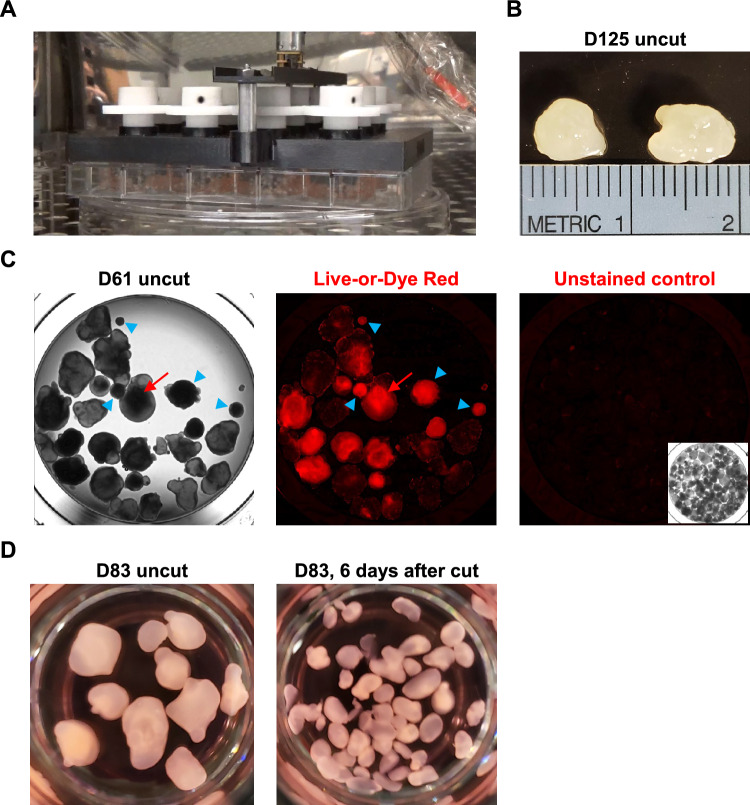
Long-term organoid culture in mini-spin bioreactors. **A** A mini-spin bioreactor with organoids. **B** Organoids cultured for 125 days reached 6–8 mm in size. **C** Examples of small dead organoids (blue arrowheads) and large organoids displaying dead regions (red arrows) that are dark in bright-field image (left) or fluoresce bright red when organoids are stained with Live-or-Dye Red (middle). Unstained organoids are shown as a control (right). Images are taken in a 24-well plate. **D** Examples of uncut (left) and cut (right) organoids imaged in a 24-well plate

Our efforts in long-term culture demonstrate that organoids can be successfully grown for 4–5 months in mini-spin bioreactors reaching 6–8 mm in size (Fig. 1A, B). However, increased organoid size causes cells in the center of organoids to receive fewer nutrients and less oxygen, leading to the formation of a necrotic core (Fig. 1C) [10]. In addition, during the first few weeks of culture, some organoids cease proliferation and undergo extensive cell death, potentially due to off-target differentiation incompatible with present culture conditions. We generally observed 18–38% of largely dead organoids, each with a characteristically small size and dark dense tissue as seen when imaged using bright-field microscopy (Fig. 1C). In this regard, organoid cutting combined with selection by size could help to eliminate necrotic organoids, used to scale up organoid culture, and make organoids more uniform in size (Fig. 1D).

### Design of organoid cutting jigs

**Fig. 2:**
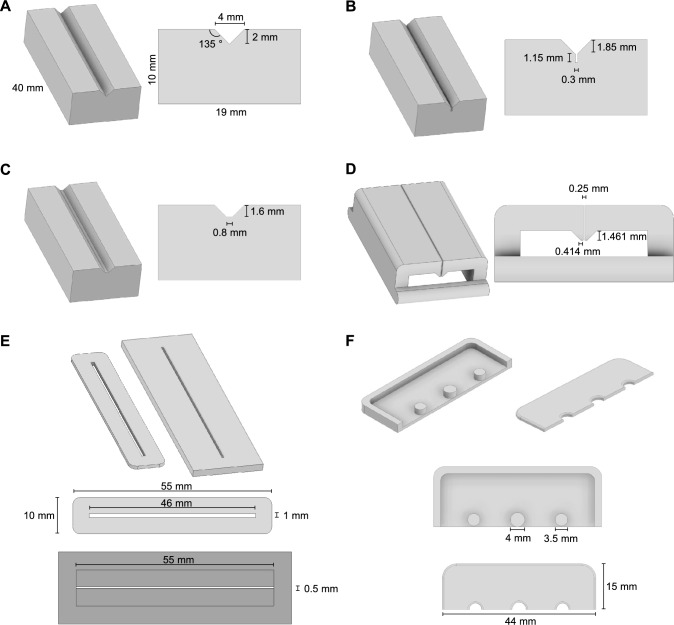
Designs of 3D printed organoid cutting devices. **A** V-shaped jig aligns organoids along the channel for cutting. **B** V-shaped jig with the addition of a 300 µm-wide channel for medium drainage away from the organoids by capillary action. **C** Flat-bottom jig, which also aligns organoids, but provides a flat cutting surface for the blade. **D** The blade guide fits around V-shaped, V-shaped with channel, and flat-bottom jigs to hold the organoids in place and direct the cutting blade to the center of the channel. **E** Racetrack jig (top left) and its blade guide (top right) align the organoids and cutting blade but enable the use of a soft cutting surface. Top-down views of the racetrack jig (top) and blade guide (bottom) are also provided. **F** A blade guard (top left) and blade guard clip (top right) provide a safe way to handle double-edged blades during the organoid cutting process. Top-down views of the blade guard (top) and blade guard clip (bottom) are also provided

3D printing, or additive manufacturing, is the production of objects based on digital models and has seen rapid adoption in the automotive, aerospace, and prototyping industries and among hobbyists over the past two decades [32]. More recently, 3D printing capabilities have greatly expanded to include the bioprinting of tissue-engineered medical products [33] and the ability to fabricate parts using ultra-high-performance materials [34]. 3D printing parts from materials that can be sterilized enables the manufacture of, for example, bioreactor impellers [27], custom culture vessels [35], and microfluidics [36], supporting rapid discovery in the bioengineering research field. To allow organoid cutting in a sterile environment, we designed and 3D printed organoid cutting jigs using BioMed Clear resin, which has low cytotoxicity and is suitable for autoclave sterilization [37]. We generated several cutting geometries and evaluated their organoid cutting performance (Fig. 2).

We designed jigs, each with a channel long enough to align about 30 organoids for simultaneous cutting. Three cutting jigs with varying 3D printed channel geometry were made: a V-shaped groove with sharp angle at the bottom (Fig. 2A), a V-shaped groove with a narrow 300 µm-wide channel at the bottom allowing medium removal via capillary force (Fig. 2B), and a V-shaped groove with flat-bottom (Fig. 2C). In all three designs, the channel is 40 mm long and, after organoid placement, a blade guide secures the organoids in place and aids accurate bisection of the organoids by the cutting blade (Fig. 2D). The V-shaped groove aligns all organoids, regardless of size, at the center of the channel. The addition of a medium collection channel to the V-shaped groove geometry allowed us to easily remove all medium, which can cause organoid movement during the cutting process. In contrast, the flat-bottom channel does not promote organoid centering as well as the V-shaped geometries, but it provides a flat surface for the blade to make full contact with the jig.

During the cutting jig design, it was hypothesized that a soft cutting surface might yield improved results over the jigs with stiff 3D printed channels. We designed a fourth racetrack jig (so called due to its resemblance to a racetrack) with a 46 mm long channel placed on a PDMS surface (Fig. 2E). We also 3D printed a blade guide for the racetrack jig. Finally, to hold the razor blades and prevent user injury during organoid cutting, a razor blade guard was designed and 3D printed (Fig. 2F).

### Cutting reduces the average organoid diameter

**Fig. 3:**
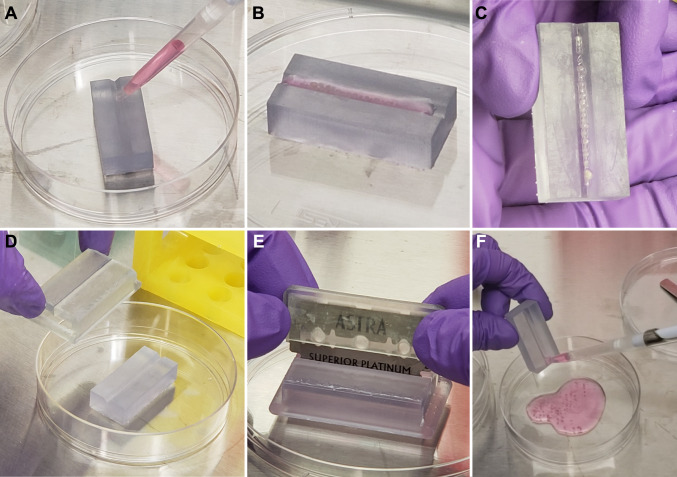
Technique for cutting organoids to enable extended culture. **A, B** Organoids are pipetted into the cutting jig channel, and the medium is removed. **C** Organoids are positioned into a line with sterile tweezers. **D** The blade guide is placed on top of the organoid-containing jig. **E** The blade is pushed through the blade guide slot to cut the organoids. **F** Organoids are flushed from the jig and collected for continued bioreactor culture

Organoids were cut using the four 3D printed geometries and compared to uncut control organoids. Briefly, organoids were collected from bioreactor culture and pipetted into the cutting jig channel (Fig. 3A, B). After removal of excess medium, sterile fine point tweezers were used to arrange the organoids in the cutting channel (Fig. 3C). The blade guide was placed on top of the cutting jig (Fig. 3D) and the blade pushed through the blade guide to slice the organoids (Fig. 3E). After removal of the blade guide, organoids were washed out of the cutting jig channel (Fig. 3F) and collected for analysis or continued culture. Additional details on organoid cutting are provided in the Methods section.

**Fig. 4:**
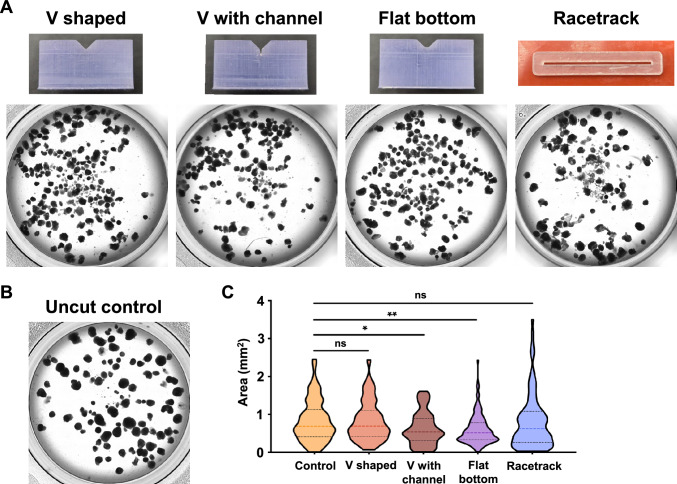
Efficacy of organoid cutting jigs. **A** Images of organoids in 12-well plates captured immediately after cutting with associated jigs show the resulting size and shape of organoids. **B** Uncut control organoids in 12-well plates. **C** The area of each organoid was measured, and data was presented as a violin plot to highlight size distribution. Colored dashed lines represent the median. Black dashed lines represent the range between the 25th and 75th percentiles. The flat-bottom cutting jig showed the most significant reduction in organoid size and the tightest grouping around the average organoid area. Images of cut organoids and controls (n = 91–148) from one representative cutting experiment were analyzed using ImageJ to determine organoid size reduction. Asterisks indicate statistical significance: *p < *0.05* **p < *0.01*, n.s. (not significant) when compared to the control

The efficacy of each cutting jig was evaluated by slicing organoids cultured for 35 days and imaging all organoid fragments collected after the process (Fig. 4A). The V shaped jig resulted in some organoids being cut, but many others remained uncut or only partially sliced, likely due to incomplete contact of the cutting blade with the bottom of the V-shaped channel. The V-shaped jig with a medium collection channel resulted in some organoids being pushed into the medium collection channel by the cutting blade. These could not be collected and were likely damaged. Most organoids were successfully cut by the flat-bottom jig, which had no usability concerns. The racetrack jig was challenging to work with because the jig was not secured to the PDMS cutting surface. Significant squishing of organoids occurred, which resulted in the area of some organoids being inadvertently increased. Uncut control organoids were also imaged and had an average area of 0.84 mm^2^ (± 0.55 mm^2^) (Fig. 4B). An analysis of organoid area post-cut (Fig. 4C) revealed that the flat bottom cutting jig resulted in the smallest average organoid area of 0.60 mm^2^, which was significantly smaller than the uncut organoids (*p* = *0.0019*). Although the V shaped jig with a medium collection channel resulted in a similar reduction in organoid size (0.63 smm^2^), the larger standard deviation and high incidence of squished organoids in the medium collection channel made it a less-than-ideal cutting geometry. Therefore, the flat-bottom jig was selected as the best organoid cutting geometry.

### Organoid cutting increases cell proliferation

Previous studies utilizing long-term organoid culture reported the formation of necrotic cores due to the limited diffusion of nutrients and oxygen, which could be alleviated by systematic organoid cutting [10, 16]. Similarly, in our studies, we also noticed the development of necrotic areas in large organoids beginning at 35-40 days of culture (Fig. 1C) [28].

**Fig. 5:**
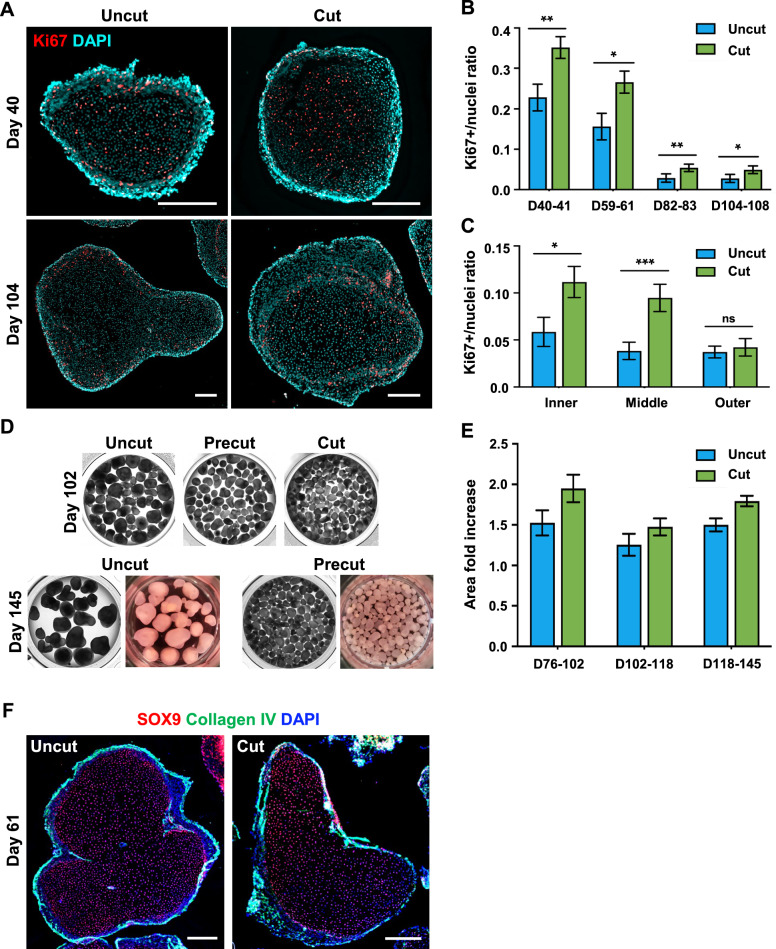
Analysis of organoid growth and cell proliferation during long-term culture. **A** Representative images of sectioned uncut and cut organoids showing Ki67+ nuclei after 40 and 104 days of culture (scale bars = 250 µm). **B** Quantification of Ki67+ nuclei in 200 × 200 μm fields within sectioned uncut and cut organoids. Data are presented as mean ± SEM. n = 2 independent experiments with n = 10 organoids/technical replicates for each group. Asterisks indicate statistical significance: *p < 0.05 **p < 0.01, ***p < 0.001, n.s. (not significant). **C** Quantification of Ki67+ nuclei localization in organoids divided into inner, middle, and outer regions. Data are presented as mean ± SEM. n = 2 independent experiments with n = 10 organoids/technical replicates for each group. Asterisks indicate statistical significance: *p < 0.05 **p < 0.01, ***p < 0.001, n.s. (not significant). **D** Representative bright-field images of intact organoids that are uncut, precut (image taken before organoid cutting), and cut. **E** Quantification of the fold increase in the size of organoids shown in D during long-term culture of uncut organoids and between cuttings for routinely cut organoids. Data from a single representative experiment are shown as mean ± SEM. **F** Uncut and cut D61 organoids immunostained for SOX9 (Sertoli cell marker) and collagen IV (seminiferous tubule marker) (scale bar = 250 µm)

Therefore, we cut organoids at three-week intervals (± 3 days), spanning a total culture period of approximately 100 days. To assess cell proliferation, we quantified Ki67+ nuclei inside uncut and cut organoids (Fig. 5A, B). After the first cutting (D40-41), cut organoids had higher Ki67+ to DAPI-stained nuclei ratios compared to uncut organoids (0.35 ± 0.03 vs 0.23 ± 0.03, respectively; *p* < *0.05*) (Fig. 5B). Similarly, after the second round of cutting (D59-61), cut organoids had higher ratios of Ki67+ to nuclei compared to uncut organoids (0.27 ± 0.03 vs 0.16 ± 0.03; respectively; *p* < *0.05*) (Fig. 5B). We found a large decrease in proliferation after the second cutting, which is likely due to cells reaching terminal stages of differentiation and expected cessation of proliferation. However, cut organoids still contained higher ratios of Ki67+ to nuclei compared to uncut for both the third (D82-83) and fourth (D104-108) rounds of cutting (Fig. 5B). Overall, these data indicate that cutting organoids results in greater cell proliferation when cultured over 100 days.

To evaluate the localization of proliferating cells, we quantified Ki67+ nuclei in distinct regions that span the inner, middle, and outer compartments of organoids at D104 (Fig. 5A, C). We found that cut organoids contained significantly higher ratios of Ki67+ nuclei in the inner and middle regions of the organoids compared to uncut (*p* < *0.05*), thus suggesting that cells located towards the inside of organoids are better able to proliferate when the organoids are cut (Fig. 5C). We found no difference in the ratio of Ki67+ nuclei on the outside of the organoid (*p* = *0.33*) (Fig. 5C).

Cut organoids demonstrated relative uniformity in size and shape compared to uncut ones (Fig. 5D). The analysis of changes in organoid size also showed the increased growth of cut versus uncut organoids (Fig. 5E). In addition, cut and uncut organoids show similar cellular and morphological organization as shown by SOX9-expressing cells in the interior (Sertoli cell marker) and encapsulation by the extracellular matrix (ECM) protein collagen IV (seminiferous tubule marker) (Fig. 5F).

### Dense organoid arrays enable high-throughput analysis

Organoid cutting improves culture conditions by increasing cell proliferation, decreasing cell death during long-term culture, and contributes to the scalability of culture and uniformity of organoids. An important component of organoid research is the identification of specific cell types and the analysis of their tissue-specific morphological organization and functionality compared to the native organ or tissue. One of the common comprehensive methods of analysis is using spatial transcriptomics tools, such as those offered by 10× Genomics Visium and Visium high-definition oligo arrays for binding RNA [20, 38, 39]. However, the high cost is accompanied by a relatively small slide capture area containing oligos measuring 6.5 × 6.5 mm in size. Because organoids are not large pieces of solid tissue that would cover the entire capture area, the positioning of multiple organoid sections on the capture area is very difficult and leads to much of the slide capture area remaining unused.

**Fig. 6:**
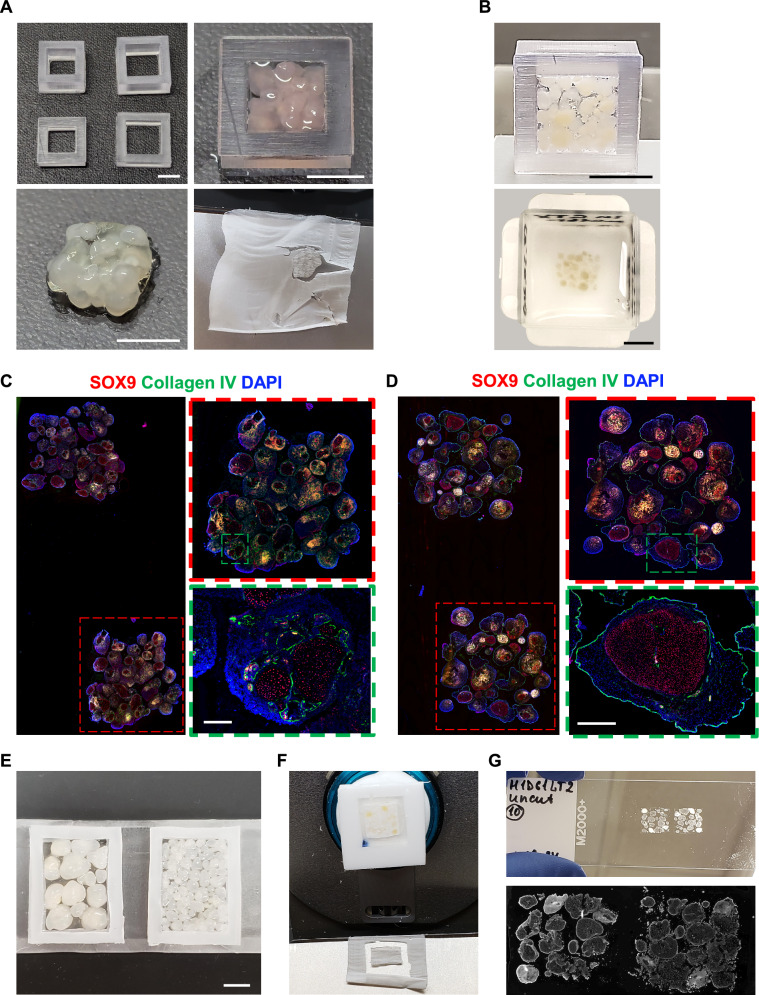
Preparation of organoids for high-density embedding, sectioning, and histological staining. **A** Variety of rigid mold sizes (top left), organoids embedded in GelMA and placed in rigid molds (top right), organoids in GelMA removed from rigid mold (bottom left), and cryosectioned organoids embedded in GelMA (bottom right) (scale bars = 5 mm). **B** Organoids embedded in Geltrex within a rigid mold (top) and subsequently frozen in OCT (bottom) (scale bars = 5 mm). **C** GelMA-embedded organoids immunostained for SOX9(Sertoli cell marker) and collagen IV (seminiferous tubule marker) and stained with DAPI (scale bar = 200 µm). **D** Geltrex-embedded organoids immunostained for SOX9 and collagen IV and stained with DAPI (scale bar = 500 µm). **E** Organoids placed in soft silicone molds (scale bar = 5 mm). **F** Organoids in soft silicone mold frozen in OCT block and cryosectioned. **G** Cryosections of organoids from soft silicone molds placed on glass slides (top) and stained with DAPI (bottom)

To maximize coverage of oligo-probed slide capture areas on Visium oligo array slides, we developed a few methods to compactly organize organoids into molds such that grouped organoids can be efficiently sectioned with minimal OCT and deposited onto slides (Fig. 6). We 3D printed molds from BioMed Clear resin to match the oligo array areas on Visium slides, placed organoids in the mold, and encapsulated them in either rigid GelMA (Fig. 6A) or soft Geltrex (Fig. 6B). Encapsulating organoids into a ECM gel allowed for their removal from the BioMed Clear mold and transfer into OCT-filled embedding molds. During cryosectioning of the OCT block, GelMA-embedded organoid sections can be easily separated from the surrounding OCT and deposited on slides. However, Geltrex-embedded organoid sections were more fragile and required mechanical removal of excess OCT. We show that both ECM gels are suitable for efficient deposition on slides and can be successfully immunostained with SOX9 and collagen IV, indicating compatibility with oligo arrays (Fig. 6C, D).

We also developed a strategy for simultaneous cryosectioning and removal of excess OCT. For this method, we utilized a soft silicone mold, which can be easily cut with a scalpel to match the desired size (Fig. 6E). In this scenario, organoids are simply mixed with OCT within the mold and pre-frozen before deposition into OCT embedding molds. During cryostat sectioning, the presence of the mold facilitates the separation of the organoid section from excess OCT (Fig. 6F), and the sections can be conveniently deposited on slides (Fig. 6G).

## Discussion

Organoid models have become widely used in drug discovery, developmental biology, and disease modeling. Their adoption has been rapid as organoids provide a platform to culture human tissues in a 3D environment while also eliminating the high costs and limitations of animal models. However, challenges associated with long-term organoid culture and subsequent analysis persist. In contrast to organs and tissues *in vivo*, organoids lack a circulatory system, and access to nutrients through diffusion is limited, which can potentially cause the development of a necrotic core, disrupted cellular organization, and overall decreased viability as organoids grow [10]. Our study shows that diminished organoid growth and the development of necrotic areas during long-term culture are likely due to restricted diffusion (Figs. 1 and 5). To alleviate issues associated with long-term organoid culture, we designed cutting jigs and optimized an organoid cutting strategy (Figs. 2, 3 and 4), which resulted in increased cell proliferation within organoids and maintained their viability and uniformity in shape and size (Fig. 5). This finding corroborates other work showing that cell viability is improved, and cell proliferation and differentiation are augmented when organoids are sliced [10]. In addition, when modeling brain cortex development, proper differentiation of specific cell types and their localization are enhanced by slicing organoids during long-term culture [10]. Cutting organoids leads to more uniformity in their shape and size and expands the culture by multiplying the number of organoids [15, 16]. In contrast to previous reports that use mechanical and enzymatic dissociation for propagation, organoid cutting retains most of the cellular organization developed during culture [10, 15, 16].

Maintaining sterility is particularly important in long-term cultures due to the significant losses associated with contamination. The cutting method developed in this work is performed in a biosafety cabinet with sterile parts and equipment, a scenario that would be very challenging to recreate with alternative methods, such as with a vibratome [10, 17]. The tools for our cutting method are inexpensive and accessible to any laboratory conducting cell culture. 3D printing a flat-bottom jig and accompanying blade guide requires approximately 21 mL of BioMed Clear resin, costing about USD $7.33. Also, the cutting technique does not require specialized equipment or expertise to perform. It can be applied to organoid or spheroid cultures of any tissue or cell type.

Organoid models permit *in vitro* recapitulation of organogenesis, but as organoids grow and meet a maximum size without cutting, they cease cell proliferation and differentiation, thus halting further maturation [10]. By cutting organoids, developmental progression can ensue, and once a specific stage is reached, cutting can be terminated. For instance, in hPSC-derived brain organoids, progenitor cells become postmitotic neurons, which represent specific cortical layers and therefore depict maturation progress [10]. In this work, we postulate that the large decline in proliferation after the second cutting may reflect cells approaching the terminal stage of differentiation, similar to *in vivo* organogenesis. Indeed, other studies show a slowdown of cerebral organoid growth after 60 days of culture [40]. Moreover, the proliferative potential of cells in organoids depends on the tissue type and cell composition (i.e., cells with stem cell properties, such as intestinal organoids, versus terminally differentiated postmitotic neurons in brain organoids) [40]. Slicing organoids in specific regions could also remove unwanted cell types and further enhance the production of sought-after tissue formation. In human brain organoids, the fusion of multiple organoids has demonstrated that cells are capable of migrating between and integrating into organoids and facilitating the formation of various regions seen in the brain [41–43]. Since cutting organoids encourages cell proliferation and interaction, cut organoids could fuse more effectively and better drive development towards organs of interest or generate multi-organoid microsystems. *In vivo*, organ growth occurs rapidly during embryonic and fetal development, but then ceases later in adult life [44]. Therefore, organoid cutting is especially important when using fetal or embryonic stem cells to model development and where their progression to the adult phase has yet to be reached.

The organoid cutting technique described in this work can be used to cut dozens to hundreds of organoids, making it useful for a variety of developmental studies and disease models. Expanding the scale at which organoid cutting can be performed would provide sufficient experimental replicas for drug screening and environmental toxicology. Potential modifications to the cutting design presented might include the incorporation of multiple cutting channels in a single cutting jig, each with its own cutting blade. The greater number of organoids that could be cut in this way might be supported by a spinning bioreactor of larger volume [45]. Our cutting method bisects organoids, generating a roughly hemispherical shape. Others have shown that organoids can be cut into four parts, resulting in a somewhat rounder shape [16]. Although we have found that cut organoids regain their spherical shape after continued bioreactor culture, modifications to the organoid cutting jig that allow two perpendicular cuts to be performed on each organoid could lead to rounder organoids immediately after cutting and improved cellular organization over time. Finally, although the racetrack jig was ultimately unsuccessful, we were unable to conclude whether soft cutting surfaces were less effective than rigid ones, and further assessment of PDMS casting would be beneficial.

A limitation of the cutting technique we have developed is that all organoids, regardless of size, are sliced simultaneously. In this scenario, large organoids will be held in place by the blade guide, while smaller organoids that do not take up the entire channel may get pushed to the side by the cutting blade and remain uncut. Several benefits could be gained by limiting cutting or continued culture to organoids in a specific size range. First, prior to initial cutting, the removal of organoids falling outside of a set size range (e.g., between 500 and 800 µm in diameter) would eliminate small and potentially dead organoids, as well as oversized organoids that may have already formed a necrotic core [46]. The remaining organoid population would have more uniform shape and growth characteristics. Repeated size exclusion at each cutting time point would reinforce the homogeneity of organoid culture over time. Alternatively, cutting only large organoids (e.g., those with diameters greater than 800 µm) might help avoid necrotic core formation while permitting smaller organoids more time to grow without disrupting their growth, cellular organization, and differentiation. Although the technique we developed is very straightforward and we believe applicable to different types of tissue-specific organoids, it remains to be validated in future studies. In addition, modifications to the organoid cutting jigs to improve the cutting efficacy of various-sized organoids can be envisaged. These could include implementation of an adjustable jig (potentially in conjunction with the sorting of organoids by size prior to slicing), the use of real-time imaging during cutting, or even an automated cutting solution that leverages machine vision. We have made our cutting jig design files available so that other researchers may further modify them. Occasionally, during cutting, organoids can adhere to the cutting blade, the blade guide, or the cutting jig. The use of an anti-adhesive coating (e.g., Sigmacote, Sigma-Aldrich) on surfaces that come in contact with organoids could help alleviate this issue. For our study, we tested numerous cutting blades and chose Superior Platinum Astra blades, which are less than 100 µm thick and more effective at cutting organoids. Other blades, including safety razor blades, cryostat blades, and brain matrix razor blades, were found to be thicker and less effective at cutting organoids. Cutting efficacy is likely a cumulative factor of blade thickness, sharpness, and cutting tip angle. An evaluation of blades by scanning electron microscopy was beyond the scope of this work, but other researchers may attempt to find alternative blades with better cutting performance.

The straightforward preparation of high-density organoid arrays shown in this work has multiple benefits for histological evaluation and high-throughput analyses. Standard histological preparation techniques for organoids result in large areas on the slide that are empty [21]. We developed multiple approaches to conveniently organize organoids for cryosectioning using rigid or soft molds and embedding in GelMA or Geltrex (Fig. 6). These methods are accessible to researchers across diverse scientific disciplines, even those without basic histological sample preparation experience. The high-density organoid arrays described in this work enable high-throughput and cost-effective workflows. In addition, this efficient technique is almost certainly a requisite for extremely costly spatial analyses of organoids, such as in situ hybridization or spatial transcriptomics [20]. For instance, specifically-sized molds can be generated and permit the arrangement of organoids such that, after sectioning, they can fit within the capture area of Visium HD slides. These techniques can also be used for the preparation of small tissue samples, for example, mouse ovaries and fish eggs. Finally, experimental and control samples can be positioned in the same plane in the mold and processed simultaneously, eliminating variability associated with the preparation of multiple slides [47, 48].

Although the cost of GelMA and Geltrex used to create our organoid arrays (Fig. 6A–D) is relatively high, these materials are widely used in bioengineering research. It is possible that less costly materials with similar thermal gelation or UV curing properties can be used instead. The rigid molds were 3D printed using Biomed Clear resin, which requires removal of the mold prior to cryostat sectioning. Future iterations of molds could be 3D printed using a softer material, such as BioMed Elastic 50A resin (Formlabs, Somerville, MA, USA), allowing for organoid cryosectioning with the mold. On the other hand, using soft silicone or PDMS molds can alleviate the need for 3D printing equipment and enable simultaneous removal of the OCT surrounding the embedded tissue (Fig. 6E, F). Furthermore, stacking multiple silicone or PDMS molds can help make thick organoid blocks for efficient downstream processing and analysis. Lastly, it is possible to generate multi-well molds to deposit multiple samples into one OCT block, which would further reduce the effects of variation in sectioning and staining and enable an entire experiment to be processed and analyzed on a single slide [47, 48].

Organoids hold an extraordinary promise for fostering advances in the fields of disease modeling, personalized medicine, and developmental biology. The organoid market and publications in the field continue to grow at an exponential rate [49–51]. However, the increasing interior hypoxia and accelerating cell death as organoids grow in size limits culture duration and organoid complexity. The organoid cutting technique we have developed reduces organoid size, mitigates necrosis, and enhances cell proliferation within organoids. Implementing this organoid cutting technique, which enables researchers to grow complex organoids long-term and scale up organoid culture, will boost the progress of the organoid research field. Furthermore, arraying and sectioning methods we have developed will facilitate high-throughput organoid assays and promote the efficient use of advanced single-cell and spatial omics analyses.

## Supplementary Information

Below is the link to the electronic supplementary material.

Supplementary file 1 (DOCX 16 KB)

## Data Availability

The datasets used and/or analyzed during the current study are available from the corresponding author on reasonable request.
