# Supplementary material for: An Efficient Organoid Cutting Method for Long-Term Culture and High-Throughput Analyses
Source: Tissue Eng Regen Med. 2025 Jun 16;22(5):675–90. doi: 10.1007/s13770-025-00731-y (PMC12209159; doi:10.1007/s13770-025-00731-y)
Supplement: Supplementary file 1 — (DOCX 16 KB) [file 13770_2025_731_MOESM1_ESM.docx]

Supplementary Information

Image analysis details and quantification of organoid size:

- Import TIFF to FIJI/ImageJ
- Invert image *(to make organoids white)*
- Circle selection, inverse selection, and delete *(to remove edge of well and outside of well)*
- Convert image to 8-bit format
- If image has significant radial variability in lighting:
  - - Duplicate image
    - Threshold each image individually *(to maintain organoid structure in either image center or image perimeter)*
    - Invert each image
    - In image thresholded to maintain organoid structure in image center, circle select where image is properly thresholded, invert selection *(to select organoids on image perimeter)*, and delete
    - Combine images using Image Calculator AND function
  - Else:
    - Threshold image
- Noise: Remove outliers [bright; radius = 10 pixels]
- Invert image
- Binary: Fill holes
- Noise: Despeckle
- Invert image
- Wand tracing select and delete remaining non-organoid objects *(utilize original images as reference)*
- Draw line tool, draw line between adjacent organoids *(to separate adjacent organoids from each other)*
- 3D Objects Counter [minimum size = 100 pixels]
- Export size of each object (pixels)
- Calculate organoid area using scale of original images (microns/pixel)
